# Detection of the East and West African kdr mutation in *Anopheles gambiae *and *Anopheles arabiensis *from Uganda using a new assay based on FRET/Melt Curve analysis

**DOI:** 10.1186/1475-2875-5-16

**Published:** 2006-02-22

**Authors:** Katrijn Verhaeghen, Wim Van Bortel, Patricia Roelants, Thierry Backeljau, Marc Coosemans

**Affiliations:** 1 Departement of Parasitology, Prins Leopold Institute of Tropical Medicine, Nationalestraat 155, B-2000 Antwerp, Belgium; 2 Departement of Invertebrates, Royal Belgian Institute of Natural Sciences, Vautierstraat 29, B-1000 Brussels, Belgium; 3 Evolutionary Biology Group, University of Antwerp, Groenenborgerlaan 171, B-2020 Antwerp, Belgium

## Abstract

**Background:**

Appropriate monitoring of vector resistance to insecticides is an integral component of planning and evaluation of insecticide use in malaria control programmes. The malaria vectors *Anopheles gambiae s.s*. and *Anopheles arabiensis *have developed resistance to pyrethroid insecticides as a result of a mechanism conferring reduced nervous system sensitivity, better known as knockdown resistance (*kdr*). In *An. gambiae s.s*. and *An. arabiensis*, two different substitutions in the *para*-type sodium channel, a L1014F substitution common in West Africa and a L1014S replacement found in Kenya, are linked with *kdr*. Two different allele-specific polymerase chain reactions (AS-PCR) are needed to detect these known *kdr *mutations. However, these AS-PCR assays rely on a single nucleotide polymorphism mismatch, which can result in unreliable results.

**Methods:**

Here, a new assay for the detection of knockdown resistance in *An. gambiae s.s*. and *An. arabiensis *based on Fluorescence Resonance Energy Transfer/Melt Curve analysis (FRET/MCA) is presented and compared with the existing assays.

**Results:**

The new FRET/MCA method has the important advantage of detecting both *kdr *alleles in one assay. Moreover, results show that the FRET/MCA is more reliable and more sensitive than the existing AS-PCR assays and is able to detect new genotypes. By using this technique, the presence of the East African *kdr *mutation (L1014S) is shown for the first time in *An*. *arabiensis *specimens from Uganda. In addition, a new *kdr *genotype is reported in *An. gambiae s.s*. from Uganda, where four *An. gambiae s.s.* mosquitoes possess both, the West (L1014F) and East (L1014S) African *kdr *allele, simultaneously.

**Conclusion:**

The presence of both *kdr *mutations in the same geographical region shows the necessity of a reliable assay that enables to detect both mutations in one single assay. Hence, this new assay based on FRET/MCA will improve the screening of the *kdr *frequencies in *An. gambiae s.s*. and *An. arabiensis*.

## Background

Malaria vector control strategies rely on the use of insecticides for the impregnation of bed nets and for indoor residual spraying. Increasing resistance of malaria vectors may have important implications for the vector control programmes, especially considering the scaling up of insecticide treated bed nets (ITNs). Hence, knowledge on changing trends in insecticide resistance is a basic requirement to guide the use of insecticides in the malaria control programmes.

Pyrethroids are the most commonly used insecticides in the fight against malaria. These insecticides modify the gating kinetics of the *para*-type sodium channels by slowing both the activation and the inactivation of the channels [1]. An important resistance mechanism against pyrethroids and DDT, known as knockdown resistance (*kdr*), has been linked to a single mutation in the *para*-type sodium channel gene in several insect species [2]. Two different *kdr *mutations have been found in the African malaria vector *Anopheles gambiae s.s*. In West Africa, knockdown resistance is linked with a mutation resulting in a leucine to phenylalanine substitution in the S6 segment of domain II of the *para*-type sodium channel (L1014F) [3]. In Kenya, however, a different mutation was found, causing a change from leucine to serine at the same amino acid position (L1014S) [4]. Recently, the leucine to phenylalanine mutation was found in *An. arabiensis *samples from Burkina Faso [5] and the leucine to serine mutation was found in the same species from Kenya [6].

In *An. gambiae s.l*. populations, the screening for the L1014S and L1014F *kdr *mutation is commonly performed using two different allele-specific polymerase chain assays (AS-PCR) [3,4]. However, these techniques rely on a single nucleotide polymorphism mismatch at the 3'-end of a primer, and can lead to unreliable results [7]. Recently, Lynd *et al*. [7] developed a Hot Oligonucleotide Ligation Assay (HOLA) for the detection of the *kdr *mutations in *An. gambiae s.s*. This technique requires four separate hot ligation reactions to genotype specimens for the East and West African *kdr *mutation [7]. In order to improve the detection of the *kdr *mutations in *An. gambiae s.s*. and *An. arabiensis *a high-throughput test is needed that is able to detect both *kdr *alleles simultaneously in one assay. Therefore, a new assay based on Fluorescence Resonance Energy Transfer/Melt Curve analysis (FRET/MCA) was developed and tested on field collected *An. gambiae s.s*. and *An. arabiensis *specimens from Uganda.

## Methods

### Mosquito collections

Mosquitoes were collected at monthly intervals from March 2001 to February 2002 in seven sites throughout Uganda (Okello, unpublished data). Three houses per site were chosen and human landing collections were done during six consecutive nights from 20.00 hr until 06.00 hr. The mosquitoes were morphologically identified using the key developed by Gillies and Coetzee [8] and were stored on silica gel.

### DNA extraction and molecular identification of species

The protocol for DNA extraction is adapted from Vythilingam *et al*. [9]. Since collections were made in the frame of a transmission study (Okello, unpublished data), heads and thoraxes of individual mosquitoes were homogenized in 150 μl blocking buffer [10] and 50 μl of blocking buffer/Nonidet P-40 (blocking buffer containing 0.5 % Nonidet P-40). These enzyme linked immunosorbent assay (ELISA) homogenates were used for mosquito DNA extraction, by adding 200 μl of a 20% Chelex solution (Biorad, Hercules, USA) to 20 μl of the mosquito homogenate. Samples were placed in a thermo-mixer at 56°C and 1,400 rpm for 30 minutes, vortexed at high speed for six seconds and placed in a thermo-mixer at 95°C and 1,400 rpm for 10 minutes. After incubation, the samples were centrifuged twice at 13,400 g for five minutes at 4°C. The supernatants containing the DNA was transferred and stored at -20°C.

The protocol used for molecular identification of the members of the *An. gambiae *complex was adapted from Scott *et al*. [11]. Genomic DNA was mixed with the primers AR (specific for *An*. *arabiensis*), AG (specific for *An. gambiae s.s*.) and UN (common for both species) in a 25 μl reaction. Amplification reactions contained 1 μL of DNA, 1.5 mM MgCl_2_, 10 mM Tris-HCl (pH 8.4), 50 mM KCl, 0.1% Triton X-100, 200 μM of dNTP's (Amersham, Buckinghamshire, United Kingdom), 80 nM of primers UN and AR, 40 nM of primer GA and 0.25 U of Silverstar DNA polymerase (Eurogentec, Seraing, Belgium). The PCR was carried out as described in Scott *et al*. [11]. The amplified products were checked on a 2% agarose gel, stained with ethidium bromide, and visualized on the Image Master VDS (Amersham Pharmacia, Uppsala, Sweden).

### AS-PCR for detection of the L1014S and L1014F kdr alleles

**Figure 1:**
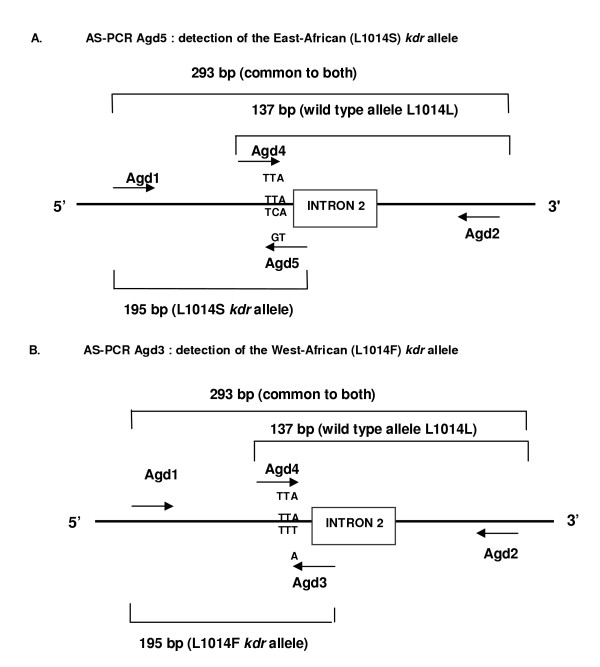
**Schematic representation of the AS-PCR Agd5 and AS-PCR Agd3. **Schematic representation of the AS-PCR Agd5, which detects the L1014S *kdr *allele (A) and the AS-PCR Agd3, which detects the L1014F *kdr *allele (B) [3-4].

The protocol used for the detection of the L1014S or L1014F *kdr *alleles was adapted from the protocols developed by Martinez-Torres *et al*. [3] and Ranson *et al*. [4]. Primers Agd1 (5'-atagattccccgaccatg-3'), Agd2 (5'-agacaaggatgatgaacc-3'), Agd3 (5'-aatttgcattacttacgaca-3') and Agd4 (5'-ctgtagtgataggaaattta-3') were used to detect the L1014F allele (AS-PCR Agd3), whereas primers Agd1, Agd2, Agd4 and Agd5 (5'-tttgcattacttacgactg-3') were used to detect the L1014S allele (AS-PCR Agd5) (Fig 1). Amplification was performed in a 50 μl reaction containing 1 μl of template DNA, 1 × Qiagen PCR buffer, 0.5 mM MgCl_2_, 100 nM of each primer, 200 μM of dNTP's, and 1 U of Taq DNA polymerase (Taq PCR core kit, Qiagen, Hilden, Germany). The cycling conditions were: initial 94°C denaturation for five minutes, 10 cycles of one minute denaturation at 94°C, 30 seconds annealing at 54°C and 30 seconds extension at 72°C, followed by 30 cycles of one minute denaturation at 94°C, 30 seconds annealing at 47°C and 30 seconds extension at 72°C, and a final extension at 72°C for 10 minutes. Amplification products were checked on a 2% agarose gel and visualized after ethidium bromide staining.

### FRET/Melt Curve analysis

Primers and probes were designed using the Meltcalc software [12]. The forward primer AgdF-ROX (5'- tggccactgttgtgaTagg-3') is labelled with ROX, 4 nucleotides from the 3'-end (Capital T), while the probe KDR-FAM (5'-tacttacgactaaatttcctat-3') is labelled with FAM, at its 3'-end. The probe complements the wild type antisense strand of the PCR product. Two non-specific g's were added to the 5'-end of the reversed primer AgdR (5'-ggtgacaaaagcaaggctaag-3') to increase the melting temperature and the GC-content of the primer.

**Figure 2:**
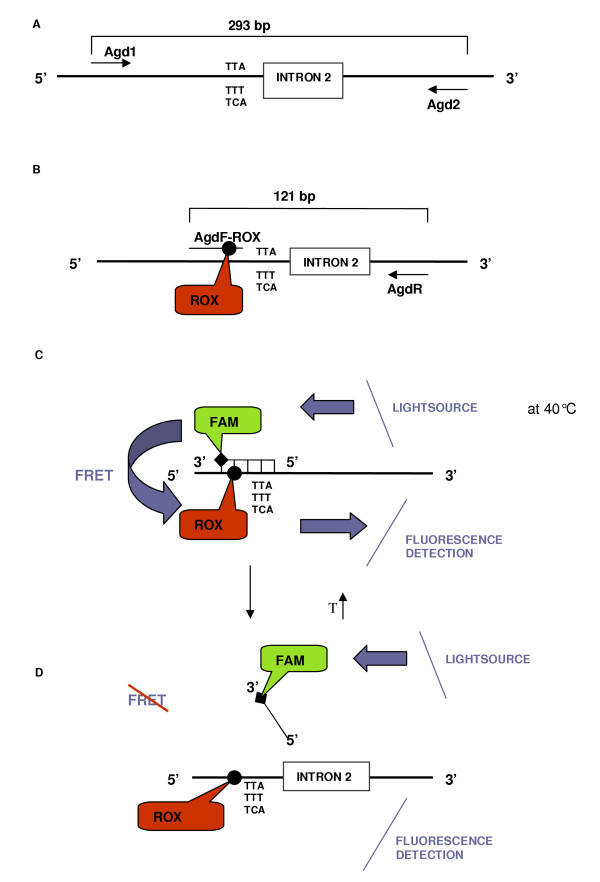
**Schematic representation of the FRET/MCA, which allows the detection of both (L1014S and L1014F) *kdr *alleles in one assay. **A. The primary PCR (primers Agd1-Agd2) results in amplification of a 293 bp fragment of the *para*-type sodium channel gene. B. During the secondary PCR, a 121 bp fragment is amplified and is labelled with ROX as the forward primer is extended. C. After amplification, the FAM-labelled probe hybridizes, and FRET starts to occur. The donor FAM-fluorophore is excited by incident light and because the ROX-acceptor is in close proximity, the excited state energy from FAM can be transferred. D. Melt curve analysis on the probe-amplicon hybrid. Progressive increase of temperature during melt curve analysis leads, at a specific temperature, to the dissociation of the probe from the amplicon. At this point, no FRET occurs and the ROX-fluorescence will decrease. During this MCA, the change in amount of fluorescence for each probe-template hybrid was plotted against the temperature and its negative derivative appeared as a positive peak.

A primary PCR was performed with a 50 μl reaction mix containing 1 μl of DNA, 1 × Qiagen PCR buffer, 1 mM MgCl_2_, 200 μM of each dNTP, 100 nM of the primers Agd1 and Agd2 and 1 U Taq DNA polymerase (Taq PCR core kit, Qiagen, Hilden, Germany). Amplification conditions were as follows: initial denaturation at 94°C for three minutes, forty cycles of one minute denaturation at 94°C, 30 seconds annealing at 47°C and 30 seconds extension at 72°C followed by a final extension of 10 minutes at 72°C (Figure 2A).

The secondary PCR assay and the MCA were performed on an iCycler with a 490/20X FAM excitation filter and a 620/30M ROX emission filter (Bio-rad, Hercules, USA). The secondary PCR was performed in a 96-well plate. Amplification reactions (50 μl) contained 1 × iQ supermix (Bio-Rad, Hercules, USA), 1 mM MgCl_2_, 500 nM of AgdF-ROX, 100 nM of AgdR and 2 μl of a 10-fold dilution of the primary PCR product resulting from amplification with Agd1 and Agd2 (Figure 2B). Cycling conditions were as follows: initial denaturation at 95°C for four minutes, forty cycles of one minute denaturation at 95°C, 30 seconds annealing at 52°C and 30 seconds extension at 72°C followed by a final extension at 72°C for eight minutes. During this asymmetric PCR, the target strand to which the FAM-labelled probe binds, was produced in excess. After amplification, the probe was added in a final concentration of 200 nM, and a melt curve was performed, consisting of 95°C for one minute, cooling to 40°C for one minute and 80 repeats heating for 20 seconds, starting at 40°C and with 0.5°C increments. During this melt curve, the cooling to 40°C will allow the FAM-labelled probe to anneal adjacent to the ROX-fluorophore of the PCR product (Figure 2C). The temperature is subsequently slowly increased, while the ROX-fluorescence resulting from FRET, is continually monitored. When the melting temperature of the probe-amplicon hybrid is reached, FRET can no longer occur, and the ROX-fluorescence will decrease (Figure 2D). Changes in the ROX-fluorescence appear as a peak on the plot of the first negative derivative of the fluorescence versus temperature function. All data were analysed with the iCycler™ iQ Optical system software version 3.0a (Bio-rad, Hercules, USA). The experiments were performed in triplicate to verify reproducibility.

Three plasmids, containing as inserts the wild type, the L1014S or the L1014F *kdr *allele, were used on each plate as positive controls. Two lines, a susceptible line  and a homozygote resistant line for the West African *kdr* allele L1014F, were  obtained from laboratory colonies of the LIN/IRD and were used to construct  the L1014L (wild type) and the L1014F plasmid, respectively. The L1014S plasmid originated from a sequenced homozygous L1014S field specimen of Uganda. The plasmids were constructed by ligation and transformation of the 293 bp PCR product resulting from amplification with primers Agd1 and Agd2, by using the Original TA cloning kit according to the manufacturer's instructions (Invitrogen, Carlsbad, California). Three millilitres of each clone was purified on a column (QIAprep Spin Miniprep kit, Qiagen, Hilden, Germany) and DNA was resuspended in 50 μl water. Two microlitres of a 100-fold dilution of the plasmid was used directly in the secondary, asymmetric PCR of the FRET/MCA assay instead of the template DNA. Plasmid and direct PCR sequencing were performed by the VIB genetic service facility (University of Antwerp, Belgium).

## Results

**Figure 3:**
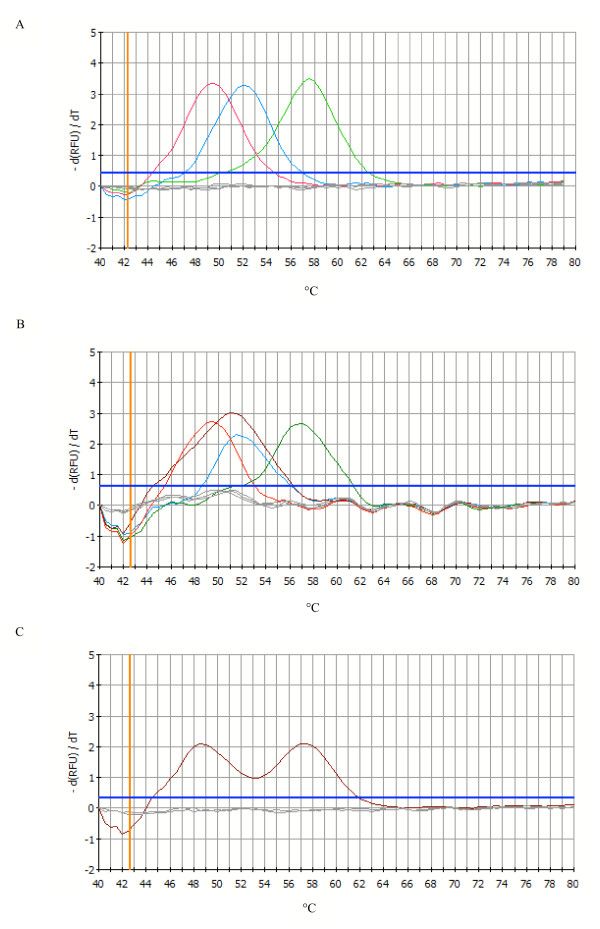
**FRET/MCA of the DIIS6 region of the *para*-type sodium channel gene of *An. gambiae s.s*. and *An. arabiensis *plotted as the first negative derivative of the relative fluorescence unit (-d(RFU)/dT) versus temperature function. **In all 3 panels, the thicker blue horizontal line denotes the threshold for background fluorescence, and the curve entirely below denotes the results for the non-template control (gray). A. Cloned sequence variants, from low to high T_m_: The L1014S allele (red), the L1014F allele (blue) and the wild type L1014L allele (green). Melting peaks were identified at 49.5°C, 52°C and 57.5°C, respectively. B. *An. gambiae s.s*. specimens, from low to high T_m_: homozygous for the L1014S allele, T_m _of 49.5°C (red) ; specimens heterozygous for both *kdr *alleles (L1014S/L1014F allele), T_m _of 51°C (dark red) ; specimens homozygous for L1014F allele, T_m _of 52°C (blue) and specimens homozygous wild type, T_m _of 57.5°C (green). C. *An. gambiae s.s*. specimens heterozygous for the L1014S/wild type (dark red) were characterised by two melting peaks of 49.5°C and 57.5°C. Heterozygous (L1014S/wild type) *An. arabiensis *specimens showed the same pattern.

Melt curve analysis of the plasmids produced three different melting curves with melting temperatures (T_m_) of approximately 49.5°C, 52°C and 57.5°C for the L1014S *kdr *allele, the L1014F *kdr *allele and the wild type allele, respectively (Figure 3A). As expected, the wild type sequence DNA showed the highest melting temperature. The existence of a nucleotide mismatch between the target DNA and the hybridization probe produced a lower T_m _than the T_m _of the wild type sequence.

The melt curve obtained for mosquitoes homozygous for the L1014F, the L1014S or the wild type allele was identical to the melt curve obtained for the corresponding plasmid (Figure 3B).

Mosquito specimens, which were heterozygous for the L1014S/wild type, were characterised by two peaks with T_m _of approximately 49.5°C and 57.5°C (Figure 3C). For the first time, two *An. arabiensis *specimens heterozygous for the L1014S/wild type mutation were found in Uganda [GenBank:DQ263749]. Four *An. gambiae s.s*. specimens produced a melting peak with a T_m _of approximately 51°C, which was different from the expected T_m _for the wild type or the mutant alleles (Figure 3B). Cloning and sequencing of the 293 bp fragment of domain II of the *para*-type sodium channel gene revealed that these specimens possess both, the L1014S and L1014F *kdr *allele, simultaneously [GenBank:DQ263748]. In theory, two peaks would be expected for these samples, one at 49.5°C and one at 52°C. Instead, these samples showed a broad peak at 51°C, due to a too small difference in melting temperature between the L1014S and L1014F *kdr *allele (Figure 3B).

**Figure 4:**
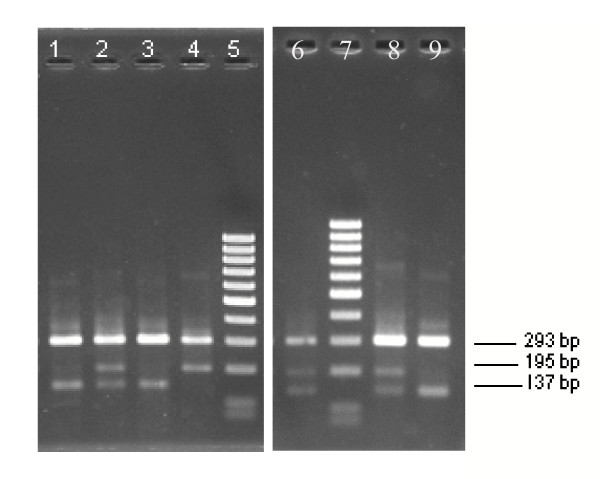
**PCR products obtained using the AS-PCR Agd5 adapted from Ranson *et al*. [4] on *An. gambiae s.s*. after separation on a 2% agarose gel. **Lane 1, Lane 3, Lane 9: homozygous wild type mosquitoes (L1014L/L1014L); Lane 2 and Lane 8: heterozygous specimens (L1014L/L1014S); Lane 4: homozygous resistant specimen (L1014S/L1014S); Lane 5 and Lane 7: 100 bp ladder; Lane 6: specimen scored as heterozygous (L1014L/L1014S) by AS-PCR, but scored as homozygous resistant (L1014S/L1014S) by FRET/MCA.

The FRET/MCA results were compared with the two AS-PCR assays for the detection of the West (L1014F) and East (L1014S) African *kdr *mutation, i.e. AS-PCR Agd3 and AS-PCR Agd5. Therefore, 290 wild-caught *An. gambiae s.s*. and 183 *An. arabiensis *specimens from Uganda were tested by use of the three detection methods. By comparing the FRET/MCA and the AS-PCR Agd5 results, 121 *An. gambiae s.s*. and 181 *An. arabiensis *mosquitoes showed the same homozygote genotype for L1014L. Both assays showed the same heterozygous genotype L1014S/wild type for 113 *An. gambiae s.s*. and two *An. arabiensis *specimens and the same homozygous genotype for L1014S for 38 *An. gambiae s.s*. mosquitoes (Table 1). The four *An. gambiae s.s*. specimens, heterozygous for both *kdr *alleles (L1014S/L1014F) were scored as homozygous L1014S in the AS-PCR Agd5. This is expected since the AS-PCR Agd5 should not detect the L1014F. Although the AS-PCR assays were adapted to give well interpretable results (Figure 4), there was discrepancy between the FRET/MCA and the AS-PCR Agd5 results for 10 *An. gambiae s.s*. specimens (Table 1). Sequence-analysis revealed that in all these cases the FRET/MCA method gave the correct result.

**Table 1:** Comparison between the FRET/MCA and the AS-PCR Agd5. The AS-PCR Agd5 only detects the East African kdr mutation (L1014S).

Species	AS-PCR Agd5	FRET/MCA			
		
		S/S	S/RE	RE/RE	RE/RW
*An*. *gambiae s.s*.	S/S	121			
	S/RE	3	113	1	
	RE/RE		6	38	4
	negative	2	2		
					
*An. arabiensis*	S/S	181			
	S/RE		2		

S: Wild type allele (L1014L)

RE: Resistant allele, East African mutation (L1014S)

RW: Resistant allele, West African mutation (L1014F)

By comparing the FRET/MCA and the AS-PCR Agd3 results, 124 *An. gambiae s.s*. and 181 *An. arabiensis *mosquitoes showed the same homozygote genotype for L1014L (Table 2). In total, 119 *An. gambiae s.s*. and two *An. arabiensis *mosquitoes were scored homozygous wild type by AS-PCR Agd3, whereas they were scored as heterozygous L1014S/wild type by FRET/MCA. This is expected since the AS-PCR Agd3, designed for the detection of L1014F, should not detect the L1014S. However, 39 *An. gambiae s.s*. samples scored by FRET/MCA as homozygous for the East African *kdr *mutation (L1014S/L1014S), were scored as homozygous susceptible by AS-PCR Agd3 (Table 2). The FRET/MCA results of these 39 *An. gambiae s.s *samples were confirmed by sequence analysis. Hence, when the AS-PCR Agd3 is performed on samples possessing the L1014S allele, primer Agd4 can mis-anneal to the L1014S allele and lead in combination with primer Agd2 to an erroneous amplification of a 137 bp PCR product, which is mis-recognized as a wild type allele. This non-specific annealing is not unlikely to occur in absence of a specific primer for L1014S, there is only one nucleotide mismatch between the second nucleotide from the 3'-end of primer Agd4 and the sequence of the L1014S allele (T-C mismatch) (Figure 1).

**Table 2:** Comparison between the FRET/MCA and the AS-PCR Agd3. The AS-PCR Agd3 only detects the West African *kdr *mutation (L1014F).

Species	AS-PCR Agd3	FRET/MCA			
		
		S/S	S/RE	RE/RE	RE/RW
*An*. *gambiae s.s*.	S/S	124	119	39	
	S/RW				2
	RW/RW				2
	negative	2	2		
					
*An. arabiensis*	S/S	181	2		

S: Wild type allele (L1014L)

RE: Resistant allele, East African mutation (L1014S)

RW: Resistant allele, West African mutation (L1014F)

Interestingly, the FRET/MCA was able to score 4 samples which were negative in both AS-PCR assays: two were homozygous susceptible and 2 heterozygous resistant for the L1014S allele. This indicates that the reliability of the AS-PCR can depend on the template concentration, while the FRET/MCA is less sensitive to changes in DNA template concentration.

## Discussion

The ability to determine the resistance status of *An. gambiae s.l*. is essential to guide the use of insecticides in the African malaria control programmes. It allows for a rational choice of insecticide to be made, based on the type and extent of resistance present. Knockdown resistance has been detected in *An. gambiae s.s*. and *An. arabiensis *[3-6]. The screening for the L1014S and L1014F *kdr *mutation, causing the knockdown resistance, is commonly performed using two different AS-PCR assays [3,4]. These assays provide a cheap mean for determining the *kdr *allele frequencies in *An. gambiae s.s*. and *An. arabiensis *populations. Recently, Lynd *et al*. [7] developed a new assay based on HOLA for the detection of the *kdr *alleles.

Here, a new high-throughput assay based on fluorescent hybridization and melt curve analysis for the detection of both *kdr *mutations, is described. Although this FRET/MCA technique requires a high initial equipment outlay, consisting of a standard PCR cycler equipped with an optical module, and requires expensive reagents, this technique offers several advantages over the existing methods.

First, the AS-PCRs rely only on a single nucleotide polymorphism at the 3'-end of the primer and can lead to unreliable results [7]. Although, the existing AS-PCR assays were adapted to obtain better interpretable results, there was still a discrepancy between the AS-PCR and the FRET/MCA results for 10 *An. gambiae s.s*. specimens (Table 1). In all these cases, the FRET/MCA method gave the correct result. Moreover, the FRET/MCA assay showed to be more sensitive and less depending on the template concentration than the existing AS-PCR assays.

Secondly, as new mutations arise at the probe-amplicon hybrid, these mutations are likely to be detected by the FRET/MCA assay. In contrast, the AS-PCR and the HOLA technique can only detect resistance alleles of known sequence. If other mutations arise, they need to be characterised at gene sequence level so that a new AS-PCR or HOLA assay can be developed, alongside the existing ones.

Finally, the major advantage of the FRET/MCA technique is its ability to screen simultaneously for both *kdr *alleles in one well by the use of only one probe. In contrast, two AS-PCR assays (AS-PCR Agd3, AS-PCR Agd5) or 4 separate HOLA reactions are required to genotype specimens for both *kdr *mutations. In addition, the FRET/MCA is designed to run in a 96-well format with integrated software to analyse the fluorescence data. In the initial development stage, samples were run in triplicate to verify reproducibility. Given the high reproducibility and ease of interpretation, up to 90 specimens can be screened for both *kdr *alleles on a single microtitre plate, allowing fast screening of the *kdr *mutations in a geographical region.

The FRET/MCA technique was applied on mosquitoes from Uganda to show its applicability in detecting *kdr *alleles in field collected specimens. The assay worked well on DNA extracts from ELISA homogenates of mosquitoes as well as on simplified extracts of one mosquito leg (data not shown). The FRET/MCA could clearly distinguish between the different *kdr *genotypes in *An. gambiae s.s*. and *An*. *arabiensis *specimens. The East African *kdr *mutation (L1014S) was found in heterozygous state in *An*. *arabiensis *specimens from Uganda. Likewise, Stump *et al*. [6] had found the same mutation in *An. arabiensis *from Kisumu, a site close to one of the study sites in Uganda. *Kdr *seems to be present in low frequency in *An. arabiensis*. However, increased insecticide pressure can increase the *kdr *frequency as was demonstrated in Kenya, where ITN introduction doubled the frequency of the L1014S *kdr *allele in *An. gambiae s.s*. [6]. This points to the fact that high-throughput methods are needed for the monitoring of the *kdr *allele.

Moreover, a new *kdr *genotype has been found in *An. gambiae s.s*. from Uganda. In two collection sites in Uganda, *An. gambiae s.s*. mosquitoes were found to possess both the West (L1014F) and East (L1014S) African *kdr *alleles, simultaneously. Interestingly, this West African *kdr *allele (L1014F) was only found in combination with the East African *kdr *allele (L1014S) in *An. gambiae s.s*. In all steps, from DNA extraction to FRET/MCA assay, appropriate negative controls were included. Moreover, the four heterozygotes originated from different localities and were extracted and tested on different days. Hence, contamination can be ruled out. The biological reason for the simultaneous occurrence of both mutations is subject of ongoing research. It is already clear that the presence of this West African *kdr *mutation (L1014F) in Uganda has important implications for the monitoring of *kdr *resistance in Africa. The reported absence of the L1014F in East African countries and of the L1014S in West African countries is probably due to the fact that in most cases only one or the other AS-PCR was used to detect *kdr*. In this way, the East African L1014S allele may perhaps been overlooked in West Africa. However, the present FRET/MCA survey clearly demonstrates that the West African L1014F allele also occurs in East Africa.

## Conclusion

The FRET/MCA assay allows the genotyping of *An. gambiae s.s*. and *An. arabiensis *specimens for both *kdr *alleles by the use of only one probe. The assay results in clearly interpretable melting peaks for the different *kdr *genotypes and works well on DNA extracts from ELISA homogenates as well as on simplified extracts of one mosquito leg. Moreover, the results show that the FRET/MCA is more reliable and more sensitive than the existing AS-PCR assays and is able to detect new genotypes. A new genotype was found in Uganda where four *An. gambiae s.s*. mosquitoes possessed both, the West (L1014F) and East (L1014S) African *kdr *allele, simultaneously. The presence of both *kdr *alleles in a same geographical region shows the necessity of a reliable assay that enables the detection of both *kdr *alleles in one assay. Hence, this new high-throughput FRET/MCA assay will improve the screening of the *kdr *frequencies in *An. gambiae s.s*. and *An. arabiensis*.

## Authors' contributions

KV developed and carried out the FRET/MCA method, carried out the AS-PCR and drafted the manuscript. WVB and MC participated in the conception of the study, revised the work critically at all stages, and substantially helped to draft the manuscript. PR carried out the vast majority of the laboratory work. TB critically reviewed the manuscript.

## References

[B1] Lund AE, Narahashi T (1983). Kinetics of sodium channel modification as the basis for the variation in the nerve membrane effects of pyrethroids and DDT analogs. Pestic Biochem Physiol.

[B2] Soderlund DM, Knipple DC (2003). The molecular biology of knockdown resistance to pyrethroid insecticides. Insect Biochem Mol Biol.

[B3] Martinez-Torres D, Chandre F, Williamson MS, Darriet F, Berge JB, Devonshire AL, Guillet P, Pasteur N, Pauron D (1998). Molecular characterization of pyrethroid knockdown resistance (kdr) in the major malaria vector *Anopheles gambiae *s.s. Insect Mol Biol.

[B4] Ranson H, Jensen B, Vulule JM, Wang X, Hemingway J, Collins FH (2000). Identification of a point mutation in the voltage-gated sodium channel gene of Kenyan *Anopheles gambiae *associated with resistance to DDT and pyrethroids. Insect Mol Biol.

[B5] Diabaté A, Baldet T, Chandre F, Dabire KR, Simard F, Ouedraogo JB, Guillet P, Hougard JM (2004). First report of a kdr mutation in *Anopheles arabiensis *from Burkina Faso, West Africa. J Am Mosq Control Assoc.

[B6] Stump AD, Atieli FK, Vulule JM, Besansky NJ (2004). Dynamics of the pyrethroid knockdown resistance allele in Western Kenyan populations of *Anopheles gambiae *in response to insecticide-treated bed net trials. Am J Trop Med Hyg.

[B7] Lynd A, Ranson H, McCall PJ, Randle NP, BlackIV WC, Walker ED, Donnelly MJ (2005). A simplified high-throughput method for pyrethroid knock-down resistance (kdr) detection in *Anopheles gambiae*. Malar J.

[B8] Gillies MT, Coetzee M (1987). A supplement to the anophelinae of Africa south of the Sahara (Afrotropical region). Publications of the South African Institute for Medical Research.

[B9] Vythilingam I, Nitiaavathy K, Yi P, Bakotee B, Hugo B, Singh B, Palmer K (1999). A highly sensitive, nested polymerase chain reaction based method using simple DNA extraction to detect malaria sporozoites in mosquitoes. Southeast Asian J Trop Med Public Health.

[B10] Wirtz RA, Sattabongkot J, Hall T, Burkot TR, Rosenberg R (1992). Development and evaluation of an enzyme-linked immunosorbent assay for *Plasmodium vivax *-VK247 sporozoites. J Med Entomol.

[B11] Scott JA, Brogdon WG, Collins FH (1993). Identification of single specimens of the *Anopheles gambiae *complex by the polymerase chain reaction. Am J Trop Med Hyg.

[B12] Schütz E, von Ashen N (1999). Spreadsheet software for thermodynamic melting point prediction of oligonucleotide hybridization with and without mismatches. Biotechniques.

